# Speculation on How RIC-3 and Other Chaperones Facilitate α7 Nicotinic Receptor Folding and Assembly

**DOI:** 10.3390/molecules27144527

**Published:** 2022-07-15

**Authors:** Ralph H. Loring

**Affiliations:** Department Pharmaceutical Sciences, Northeastern University, 360 Huntington Ave., Boston, MA 02115, USA; r.loring@northeastern.edu

**Keywords:** cys-loop receptors, multimeric protein assembly, intracellular domain, molecular modeling

## Abstract

The process of how multimeric transmembrane proteins fold and assemble in the endoplasmic reticulum is not well understood. The alpha7 nicotinic receptor (α7 nAChR) is a good model for multimeric protein assembly since it has at least two independent and specialized chaperones: Resistance to Inhibitors of Cholinesterase 3 (RIC-3) and Nicotinic Acetylcholine Receptor Regulator (NACHO). Recent cryo-EM and NMR data revealed structural features of α7 nAChRs. A ser-ala-pro (SAP) motif precedes a structurally important but unique “latch” helix in α7 nAChRs. A sampling of α7 sequences suggests the SAP motif is conserved from *C. elegans* to humans, but the latch sequence is only conserved in vertebrates. How RIC-3 and NACHO facilitate receptor subunits folding into their final pentameric configuration is not known. The artificial intelligence program AlphaFold2 recently predicted structures for NACHO and RIC-3. NACHO is highly conserved in sequence and structure across species, but RIC-3 is not. This review ponders how different intrinsically disordered RIC-3 isoforms from *C. elegans* to humans interact with α7 nAChR subunits despite having little sequence homology across RIC-3 species. Two models from the literature about how RIC-3 assists α7 nAChR assembly are evaluated considering recent structural information about the receptor and its chaperones.

## 1. Introduction

Transmembrane protein folding and assembly is not well understood even though cell surface proteins constitute some 60% of drug targets [1], and the majority have transmembrane domains (TMs). Alpha7 nicotinic acetylcholine receptors (α7 nAChRs) are pentameric ligand gated ion channels with identical subunits having four TMs (M1-M4) that fold and assemble in the endoplasmic reticulum (ER) before being further processed and transported to the cell surface [2]. α7 nAChRs are members of the Cys-loop receptor family of pentameric proteins [3] that include 5HT3, GABA_a_, and glycine receptors. Cys-loop refers to a highly conserved 13 amino acid loop located between a disulfide bond formed by two conserved cysteine residues in the N-terminal. Cys-loop receptors have at least three conformations including a “resting” state and “desensitized” form with closed channels and an “open” state in which the channel conducts ions [4]. In many cell types, α7 nAChRs require specialized chaperones such as Resistance to Inhibitors of Cholinesterase 3 (RIC-3 [5]) or Transmembrane Protein 35A (TMEM35a, also known as Nicotinic Cholinergic Receptor Regulator or NACHO) [6] to properly fold and assemble. α7 nAChRs also show great therapeutic potential [7,8,9].

Until recently, homology with other Cys-loop receptors (e.g., [10,11]), NMR studies on TMs (e.g., [12]) and homology with molluscan acetylcholine binding proteins (e.g., [13,14,15]). provided what structural information was available about α7 nAChRs. Far less was known about chaperone structures. In April 2021, Noviello et al. [16] (and later Zhao et al. [17]) published human α7 nAChR structures determined by cryo-EM and found a novel structural feature, a “latch” helix after α7 M4 laying on the extracellular surface of the membrane. This latch helix is currently unique among Cys-loop receptors. These studies also demonstrated conformational changes between the different receptor states (e.g., resting, open and desensitized). More recently, nuclear magnetic resonance (NMR) and electron spin resonance (ESR) studies revealed the highly flexible structure of the α7 nAChR intracellular domain (ICD), making these the first Cys-loop receptors with structural information about this region [18]. Meanwhile, progress in protein structure prediction has improved dramatically. In July 2021, Jumper et al. [19] released the source code for AlphaFold2, an artificial intelligence-based software that has out-performed any other program in the biennial Critical Assessment of protein Structure Prediction (CASP) competition. A companion paper [20] on the human proteome announced a database of predicted protein structures by Alphafold2 (https://alphafold.ebi.ac.uk/ (accessed on 21 July 2021)) that included (at that time) predicted structures for RIC-3 from six species, α7 nAChR structures for the same six species and NACHO structures for five of the six. (Since then, the database has expanded at the time of this writing to include α7 nAChR structures from four more species, six more RIC-3 structures including a Xenopus species [21], and three more NACHO structures, but these species do not overlap well.) The purpose of this review/analysis is to assess the current literature about how these proteins interact to help fold and assemble α7 nAChRs with a special emphasis on the actions of RIC-3. A central mystery about RIC-3 is how a family of intrinsically disordered proteins with little sequence homology across species acts to assist folding and assembly of α7 nAChR subunits from multiple species. Wang [22] and coworkers hypothesized that RIC-3 acts to pull individual pre-folded α7 nAChR subunits together through dimerization of its coiled-coiled domains, but this hypothesis precedes the discovery of NACHO. More recently, Kweon et al. [23] hypothesize that NACHO interacts with the molecular machinery involved in the early stages of α7 nAChR insertion into the ER membrane, while RIC-3 assists later in folding the subunits prior to assembly. This review examines the plausibility of these hypotheses considering the new structural information. Also, although this review focuses on the interactions between α7 nAChRs, RIC-3 and NACHO, many other proteins including Bcl2, calnexin and prototoxins help regulate the folding, expression, and degradation of these receptors [24,25,26,27]. These will be mentioned where appropriate.

## 2. Results and Discussion

### 2.1. α7 Receptor Structure

**Figure 1:**
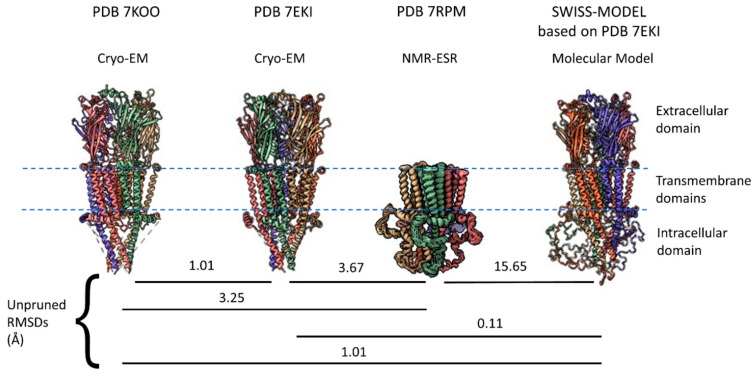
Four views of the human α7 nAChR structure as ribbon models. PDBs 7KOO and 7EKI are structures determined by cryo-EM and are missing major portions of their ICDs (dotted lines). PDB 7KOO has α-bungarotoxin bound (removed in this figure), while 7EKI is an apo-form with no bound ligands. PDB 7RPM is an ensemble of NMR-ESR structural solutions that show good homology with the regions of the cryo-EM data represented (using 7RPM A1.1 as representative data). SWISS-MODEL software generated the right-most structure based on homology with PDB 7EKI. Matchmaker [49] calculated the RMSD values between model pairs as shown.

Cys-loop receptors have three major regions for each monomeric subunit: An extracellular domain (ECD) that, for α7 receptors, includes a binding site for the snake toxin α-bungarotoxin located at the interface between subunits, four TMs (M1–M4) in the transmembrane region, and an intracellular domain (ICD) dominated in most eukaryotes by a large intracellular loop located between M3 and M4. The ECD for cys-loop receptors is the sequence between the N-terminal (after the signal peptide is removed) up to M1 and then a small extracellular loop between M2 and M3. All eukaryotic cys-loop receptor ECDs have an alpha helix and ten beta sheets in a characteristic folding pattern before M1. Until recently, acetylcholine binding proteins from mollusks served as the best homology model for the α7 nAChR ECD [13,14,15] and other cys-loop receptors provided homology for the rest of the molecule. However, in the past two years, two cryo-EM studies [16,17] provided major structural information for all three regions (Figure 1). The 7KOO PDB represents the “resting state” of human α7 nAChR but comes with α-bungarotoxin bound at all five subunit interfaces (removed in Figure 1) [16], while the 7EKI PDB [17] represents a true apo-form of the receptor. The two models show very similar structures with a root mean square deviation (RMSD) of 1.01 Å between them. However, the cryo-EM models lack a major part of the ICD, a region that had not been previously resolved in any eukaryotic cys-loop receptor. Earlier this year, an NMR-ESR study provided a model for the complete ICD in a construct that lacks the ECD [18]. Thus, in a very short time, α7 nAChRs has gone from being one of the least studied cys-loop structures to arguably one of the best studied.

Prokaryote pentameric receptors have very small ICDs, with the loop connecting M3 to M4 being less than 15 amino acids. Additionally, these receptors lack the disulfide bond creating the cys-loop in the ECD [28,29,30,31] and so can be viewed as precursors to cys-loop receptors. The best studied pentameric prokaryote receptors are from Gloeobacter violaceus (Glvi) [28] and Erwinia chrysanthemi (Elic) [30,31,32], both of which have seven amino acids in the M3-M4 loop. Even shorter prokaryotic M3-M4 loops are predicted [33]. In contrast, the glutamate anionic pentameric receptor subunit Glc-1 of Caenorhabditis elegans has 79 amino acids as one of the smallest eukaryotic ICDs (Uniprot G5EBR3) [34,35], while the Glc-2 subunit has an even smaller ICD with 74 amino acids (Uniprot Q17328). Mutations and substitutions show the importance that eukaryotic ICDs play in receptor localization and clustering [36,37], and ICDs play other important roles in receptor desensitization and signaling (for additional articles and reviews, see [38,39,40,41,42,43,44]). Eukaryotic ICDs can often withstand deletions, substitutions, and insertions without drastically affecting the assembly, trafficking and/or function of cys-loop receptors [45,46,47,48,49].

**Figure 2:**
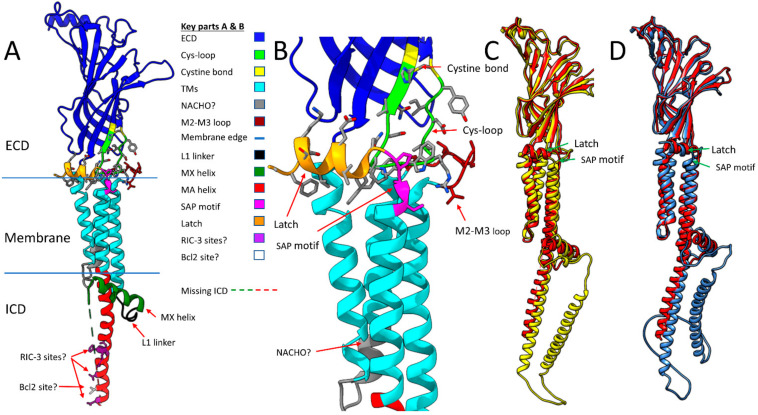

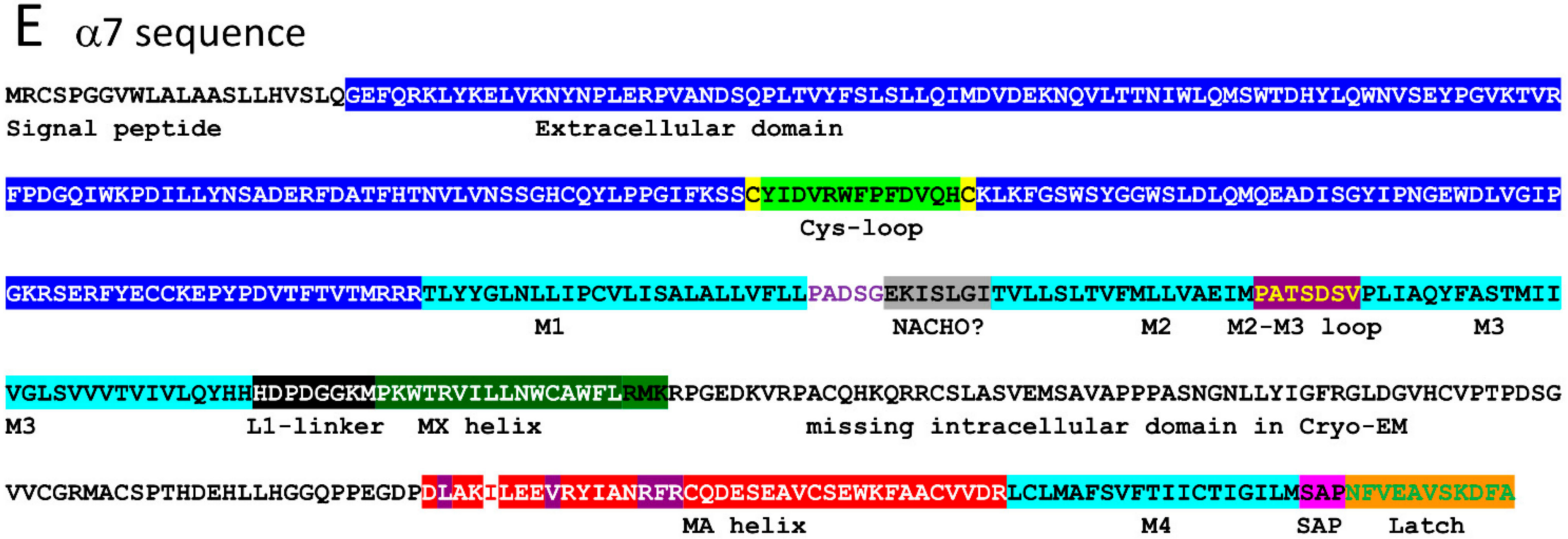
Structure of a single human α7 nAChR subunit from cryo-EM (PDBs 7EKI and 7KOO—Panels (**A**,**B**)) and comparisons across species based on AlphaFold (AF) predictions (Panels (**C**,**D**)). Panel (**E**) is a linearized version of the α7 sequence with coloring and labels as in panels (**A**,**B**).

The human α7 nAChR has 152 amino acids in its M3–M4 intracellular loop (Uniprot P36544, 153 amino acids according to NCBI NP_000737.1, and 150 according to PDB 7EKI). The M3–M4 ICD includes an eight amino acid linker after M3 (called L1 in serotonin 5HT3a receptors [50]), an MX helix located on the intracellular surface of the membrane, a largely disordered region (the missing ICD portion in cryo-EM, called Loop L by Bondarenko et al. [18]) and an MA helix that is continuous with M4 (Figure 2A). Eighty-three amino acids in the ICD (16.5% of α7 nAChR structure) remain unresolved by cryo-EM or X-ray crystallography even when large, structured proteins are inserted (e.g., *E. coli* cytochrome B562 with four alpha helices inserted into α7 nAChR [16]). Equivalent ICD regions had not been previously resolved for other Cys-loop receptors and appear largely disordered [51,52]. Therefore, the NMR-ESR data suggesting that the ICD of an α7 nAChR construct lacking the ECD (“TM + ICD” construct [18]) is relatively ordered seems incongruous with the results of other methods (Figure 1, PDB 7RPM). An ensemble of fifteen ICD structures determined by NMR and ESR differ from each other with RMSDs of no more than 3.7 Å (Average = 2.03), suggesting a relatively stable structure. For comparison, the RMSD calculated between the “resting state” of a single human α7 nAChR subunit (PDB 7KOO—blocked with α-bungarotoxin) and the human α7 “open state” (PDB 7KOX—treated with the agonist epibatidine and the positive allosteric modulator PNU120596) is 6.4 Å, suggesting that functional conformational changes induced by agonist in the rest of the receptor exceed the structural disorder in the α7 nAChR ICD found by NMR-ESR. The NMR data does suggest that the regions connecting the missing portions of the ICD loop to the MX and MA helices are very flexible and this may account for the discrepancy. The 7RPM “TM + ICD” construct assembles into pentamers and is functional, as ivermectin, an α7 nAChR positive allosteric modulator [53], activates the channel and PNU120596 potentiates this effect. Furthermore, this missing loop L has a secondary structure containing three short alpha helices according to the NMR data. Also, the TMs and MX and MA helices of the NMR 7RPM PDB (model A1.1) align well with similar structures in PDBs 7KOO and 7EKI from cryo-EM data (Figure 1), with RMSDs of 3.25 and 3.67 Å, respectively. In contrast, a molecular model from SWISS-MODEL based on PDB 7EKI randomly assigns the missing ICD structures and has an RMSD of 15.65 Å compared with model A1.1 of PDB 7RPM (NMR data), even though the rest of the model fits very well compared with the cryo-EM data (Figure 1).

The NMR data in PDB 7RPM suggests that Loop L anchors itself to the MA helix, thereby increasing its stability, but it is possible that including the ECD in the structure might change this relationship. On the other hand, the Swiss Model represents the other extreme, with Loop L being completely disordered. The truth may lie somewhere in the middle, and we await NMR evidence for a complete α7 subunit structure.

Figure 2 shows some important features that are resolved in all α7 nAChR models (Figure 2A,B) and compares representative structures of a single α7 nAChR subunit from invertebrate α7 homologues predicted by AlphaFold against the structure derived from PDB 7KOO (Figure 2C,D). All models show the M2–M3 extracellular loop and the latch (Figure 2B) surrounding the tip of the cys-loop, but also predicts that both the worm and fly intracellular domains have longer MA helices than human α7 and an additional alpha helix in the L loop (Figure 2C,D, respectively). However, the “latch” of *C. elegans* ACR-16 (the major worm α7 homolog) is too short to make a proper helix. These predictions await experimental verification. The regions between the end of M4 and the beginning of the latch have the same ser-ala-pro (SAP) motif in all six species, even though the latch sequence in the invertebrates is quite different than that observed in vertebrates. This led to a search for sequence homology in this region between α7 homologs across species.

Panel A of Figure 2 shows the main structural features from human PDB 7EKI discussed above including the extracellular domain (ECD-Blue), the cys-loop (green), the cystine disulfide bond that forms the cys-loop (yellow), the transmembrane domains M1–M4 (TMs-cyan), the intracellular MX helix (dark green), and the intracellular MA helix (also known as preM4 [54]-red). Other regions not yet discussed include the region of M2 (residues 260–267) that when substituted with the 5HT3 sequence does not require NACHO for assembly (NACHO? gray) [23], the M2-M3 loop (pink), the L1 linker between M3 and MX (black), the Ser-Ala-Pro SAP motif (magenta) and the extracellular Latch helix (orange) [16]. Also shown in purple with amino acid side chains are the five residues (L433, V440, R446, F447, and R448, numbering as in 7EKI) that when mutated to alanine in rat α7 do not need RIC-3 for assembly (RIC-3 sites? [55,56]) and the Ile residue 436 (white with side chain, I413 in PDB 7KOO) that when mutated to alanine allows assembly without the enhancing effects of Bcl2-like proteins (Bcl2 site? [54] but expression remains enhanced by RIC-3). The green and red dotted line (missing ICD Loop L) between the MX and MA helices represents 83 amino acids unresolved by cryo-EM in the intracellular loop. Panel B shows a closeup of the latch, cys-loop, and M2-M3 regions of the subunit with amino acid side chains. Panels C and D compare the alignments of human α7 PDB 7KOO (red) with *C. elegans* ACR-16 (UniProt P48180, yellow) and Drosophila α7 isoform E (UniProt E1JJR2, light blue). The RMSDs for these structures are 2.20 Å across 391 atom pairs and 2.47 Å across 382 atom pairs, respectively. AlphaFold predicts the SAP motif and Latch helices are structurally conserved across all of these species including zebrafish, mouse, and rat α7 subunits (not shown), although the C-terminal of *C. elegans* is too short to form a helix. It also predicts that Loop L has an additional helix for both worm and fly α7 as shown. Panel E shows a linearized view of the human α7 sequence with coloring and labeling similar to Panel A.

**Figure 3:**
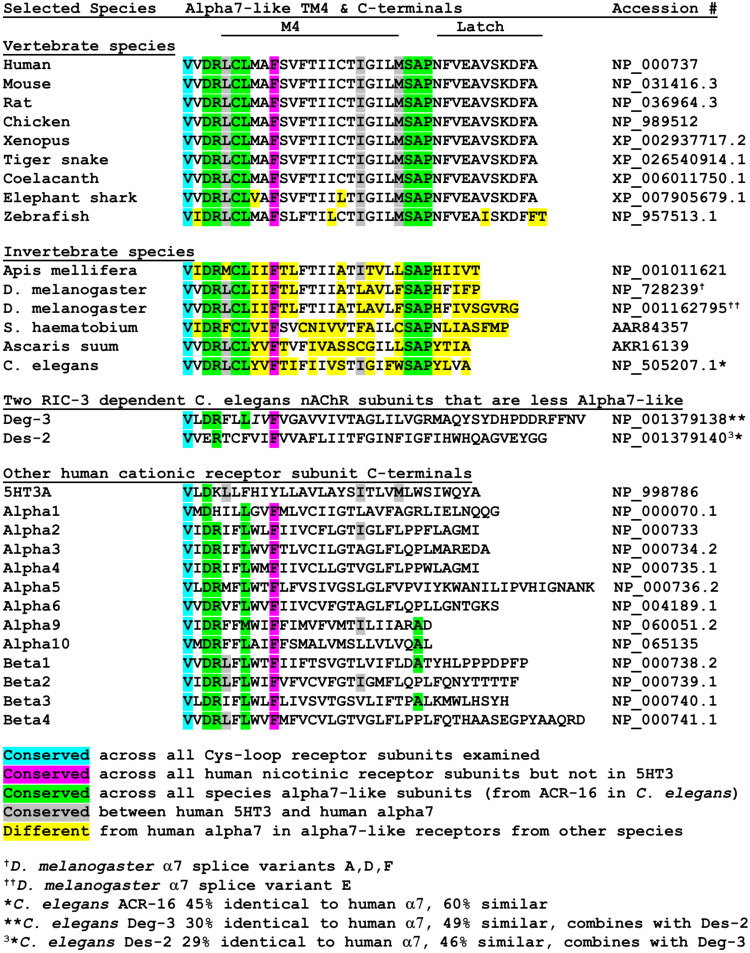
Conservation of α7 C-terminal sequences across selected species. Sequences alignment starts at V466 (cyan) in human α7 nAChR, which is conserved in all Cys-loop receptors examined. A full explanation is in the text.

The sequence of the latch helical structure currently unique to α7 nAChRs [16] is highly conserved across a variety of vertebrate species (Figure 3). Of nine vertebrate species examined, only zebrafish α7 show any sequence deviation in the latch helix and there is almost total conservation across transmembrane domain M4 as well. In contrast, sequences of six invertebrate α7 nAChRs (including two splice variants of Drosophila α7) show virtually no sequence conservation compared to human α7. Instead, a DRXCL motif located at the beginning of M4 and a ser-ala-pro (SAP) motif located just before the latch sequences are conserved across both vertebrate and invertebrate α7 sequences shown in Figure 3.

Figure 3 color-codes different amino acids in the M4 and C-terminals based on the extent of homology across a random selection of species and types of cys-loop receptors. Cyan shows a single valine residue at the human α7 position 466 conserved across all cys-loop receptors examined. Hot pink shows a single Phe at position 475 found in all nicotinic receptors examined but not in mouse 5HT3A receptor. Green shows residues conserved among all α7-like receptors starting at ACR-16 in *C. elegans*. Gray shows residues conserved between human α7 and mouse 5HT3A, while yellow shows residues that differ from human α7 in other α7-like subunits. A more thorough examination of α7 and non-α7 sequences is needed, but the SAP motif may become a diagnostic feature for α7-like receptors much like the term Cys-loop defines a subfamily of pentameric ligand-gated ion channels. For instance, recent data from three hookworm [57]. and two whipworm [58,59] species show SAP motifs in the respective ACR-16 subunits. In contrast, the DRXCL motif is conserved in some invertebrate non-alpha7 receptors (e.g., the Apis mellifera α6 subunit, NP_001073564.1 [60]) and is not unique to α7 nAChRs. In addition, the DRXXL motif is very common in other non-α7 subunits in human nAChRs (Figure 3).

*C. elegans* ACR-16 shows similarities to human α7 in that it assembles as a homomeric pentamer and has 60% sequence similarity (45% identity) according to Clustal omega. ACR-16 is the founding member of a large family of related *C. elegans* nAChRs [61,62,63]. However, the SAP motif is not conserved in other members of the ACR-16 sub-family [64] such as ACR-19. Unlike vertebrate α7 nAChRs, the α7-like ACR-16s of *C. elegans* [65] and the worm Ascaris suum [66] are insensitive to alpha-bungarotoxin but do respond to acetylcholine, nicotine, and other agonists (but the blood fluke Schistosoma ACR-16 may bind alpha-bungarotoxin [67]). In contrast, Deg-3/Des-2 are subunits in a different subfamily of *C. elegans* nAChRs [68] that have no vertebrate homologs, form obligatory heteromeric nAChRs [69,70], have less similarity to human α7 nAChRs than ACR-16, and lack the SAP motif (Figure 3). Therefore, even though invertebrate ACR-16 receptors show different pharmacology compared to vertebrate α7 nAChRs, they seem to be the most closely related.

Although the M4 TM is located some distance away from the M2 receptor ion channel, it plays an important role in cys-loop receptor function. M4 is the TM with the greatest exposure to lipids [71], and there is good evidence that this region plays an important role as a lipid sensor in muscle receptors [72,73,74]. Alanine mutations of M4 amino acids alter function in 5HT3 [75], α4β2 [76] and α7 [77] receptors when expressed in oocytes or HEK cells. Interestingly, in four of the thirteen α4β2 M4 locations where alanine substitutions block receptor function, co-expression with NACHO and RIC-3 rescued function [76] suggesting that these amino acids are important for receptor folding and assembly. Noviello et al. [16] performed mutations on α7 M4 to gain insights into the function of the latch region as well as producing a Strep-tagged version of the receptor for purification (along with other substitutions to try to stabilize the ICD or a Yellow Fluorescent Protein to monitor expression). Adding the Strep-tag to the C-terminal showed similar function to wild-type receptor, but the single channel conductance was slightly increased. The mutation of P468A or the deleting of A467 in the SAP motif blocked function but did not interfere with trafficking or surface expression, suggesting that the SAP motif has some role in receptor function. Similarly, replacing the α7 C-terminal with that of α4 (resulting in a SPP motif) blocked function. However, removing the latch helix without disturbing the SAP motif resulted in functional receptors. Taken together, these data suggest that the SAP motif is important for α7 nAChR function and may help to explain why this sequence is preserved across many species. Also, as will be discussed below, M4 and its attached latch region is the α7 structure most likely to interact with the TMD of RIC-3.

### 2.2. How What We Know about Muscle Nicotinic Receptor Assembly Informs How We Think α7 Receptors Assemble

Although we now know much more about the final assembled structure of α7 nAChRs, we have little information about the individual steps required to get the subunits folded and assembled into pentamers. The best current models for nAChR assembly are from muscle nicotinic receptors consisting of two α1s and one each of β1, γ (or ε in adult), and δ subunits per pentamer. Muscle receptors are also the gold standard for nAChR structural information. Unwin and colleagues carried out classical EM studies of hemi-crystalline arrays of Torpedo electric organ muscle receptors [78,79] that preceded current cryo-EM methods [80], but a large portion of the intracellular domain for muscle receptor remains unresolved by any method [51,80]. All muscle subunit chains insert themselves into the ER membrane in a cell-free system, but those subunits do not assemble into a state that binds α-bungarotoxin [81]. Muscle receptor synthesis starts with signal recognition particles binding to the nascent subunit proteins as they emerge from the ribosome followed by cleavage of the signal peptide in the ER lumen [82]. Pulse-chase experiments established that muscle receptor subunits fold in the ER, that α-bungarotoxin binding develops at the interface of αγ and αδ subunits [83,84,85], and that the receptors are assembled into pentamers before exiting the ER to the Golgi [86]. Two different models suggest either a heterodimer model of αγ and αδ dyads followed by insertion of β or a formation of γαβ triads followed by sequential insertion of the α and δ subunits to form the pentamer (see [2,84,85] and references therein). In either case, the final subunit order is δ–α–γ–α–β around the pentamer [80,87]. Mutational analysis suggests that the cys-loop disulfide must form, and asparagine glycosylation (Asn 141) must occur before α-bungarotoxin can bind to α subunits [88,89,90,91]. Curiously, mutation of the adjacent cysteines (Cys 192–193) that define an α-subunit does not prevent toxin binding [88], even though a disulfide bond between these residues is required for high affinity binding of agonists [92]. Furthermore, palmitoyl acyl transferases attach lipids (usually palmitate) to cysteines located on muscle receptor subunits and blocking this reaction decreases receptor surface expression in muscle or fibroblast cell lines [93,94]. Two non-selective chaperones, heavy chain binding protein (BIP) [95,96] and calnexin [97,98,99], interact with individual subunits before toxin binding develops, but not afterwards, and several steps involve changes in antibody binding to subunits (e.g., mAb41 and mAb 68 [100,101]). Evidence suggests that bungarotoxin binding develops in the ER, usually after at least partial oligomerization of the subunits ([83] and references therein). This inefficient process clearly involves multiple steps.

Since α7 subunits are identical unless manipulated, there is no easy way to determine any order in how the subunits come together to form the pentamer. (Note, however, that α7 subunits can assemble in heteromeric pentamers with β-subunits [102,103]). Cell surface α-bungarotoxin binding and electrophysiology are two methods showing that α7 pentamers are formed and inserted into the plasma membrane. A similar approach is the fluorescent detection of calcium flux through α7 receptors (usually in the presence of PNU120596) [6,104,105,106,107]. More recently, FRET (fluorescence resonance energy transfer) between two different fluorescent proteins encoded into α7 ICDs allowed the direct measurement of α7 subunit associations in the ER [46]. Another approach could be to use “electrical fingerprinting” in which the co-expression of subunits with mutations that allow either high or low-conductance channels allows the decoding of the number of agonist binding sites necessary to activate α7 pentamers [108], but this also requires assembly and insertion in the cell membrane. Chimeric α7-5HT3a receptors offer clues as to what α7 nAChR sequences influence receptor expression. Eisele et al. [109] showed that a chimera formed between α7 nAChR and serotonin 5HT3 receptors at V201 produced a functional channel that expresses much better than unmodified α7 nAChRs. Gee et al. [110] investigated whether inefficient α7 expression could be due to specific amino acid sequences by substituting other α7 regions with 5HT3a sequences. Other groups have made similar substitutions [111,112] or with sequences from other receptor subunits [113,114,115]. However, unlike muscle nAChRs, which offer a choice of antibodies that are sensitive to receptor conformation [100], α7 nAChR antibodies are problematic ([116] and references therein) and so far have not been so useful for studying α7 folding and assembly.

### 2.3. RIC-3 & NACHO Chaperone Effects and Structures (or Lack Thereof)

α7 nAChR mRNA injected into Xenopus oocytes generates functional receptor channels [117], but attempts at heterologous expression of these receptors in cell lines were problematic and highly cell-type dependent [118,119,120,121]. A mutational analysis found RIC-3 in a screen that allows *C. elegans* survival after exposure to aldicarb, an otherwise lethal acetylcholinesterase inhibitor [122]. Millet Treinin’s group discovered that RIC-3 co-injection enhances rat α7 nAChR and *C. elegans* Deg-3/Des-2 expression in oocytes but had no effect on *C. elegans* glutamate or GABA receptors [123]. RIC-3 is an intrinsically disordered ER-resident protein [70], and is conserved but with variable sequences and sizes across multiple species [70] (see below). RIC-3’s major structural features include an initial TM, which in some species is cleaved as a signal peptide [22,124], a strand or loop in the ER lumen, a second TM (referred to in this review as the putative TMD), a cytoplasmic linker followed by at least one coiled-coil domain, and then a highly variable C-terminal tail [125,126]. RIC-3 has pleotropic effects against Cys-loop receptors: Although RIC-3 increases rat α7 nAChR and *C. elegans* ACR-16 and Deg-3/Des-2 expression, it often decreases 5HT3 and α4β2 receptor expression [125] depending on the expression system. RIC-3 affects many steps in nAChR expression including the stability of unassembled nAChR subunits, the assembly of subunits to form pentamers, and the trafficking of assembled receptors as they leave the ER ([127] and references therein).

GH4C1 cells readily allow α7 nAChR expression [121,128,129,130], which raises the possibility that this cell line endogenously expresses RIC-3, as found in other cell lines, such as SH-SY5Y and some strains of PC12 cells [131]. However, knocking down RIC-3 in GH4C1 cells with shRNA had no effect on the cell line’s ability to express α7 nAChRs [129], suggesting that these cells have additional chaperones. Gu et al. [6] performed a non-biased screen of 3880 ER-resident genes and found a large response to TMEM35A (Transmembrane protein 35A), which they renamed NACHO for Nicotinic Acetylcholine Regulator. NACHO is a small 18 kD, highly conserved, ER-resident protein with four TMs that also increases the expression of other nicotinic receptors (α4β2, α6), but not non-nicotinic ligand-gated ion channels such as 5HT3 or GluA1 [6,130]. Knocking out NACHO eliminates α-bungarotoxin binding in mouse brain [6,130,132], while preliminary evidence suggests that knocking out RIC-3 only diminishes binding [132]. NACHO folds in a pattern reminiscent of a tetraspanin [133] according to AlphaFold, even though the sequence is different. Perhaps coincidentally, another tetraspanin-like-folding protein with no sequence homology is stargazin (Gene: CACNG2), the founding member of TARPs (transmembrane AMPA receptor regulatory proteins) that not only act as an ER chaperone for glutamate AMPA (alpha-amino-3-hydroxy-5-methyl-4-isoxazole propionic acid) receptors, but are also necessary for trafficking and play important regulatory roles in AMPA channel function [134,135].

**Figure 4:**
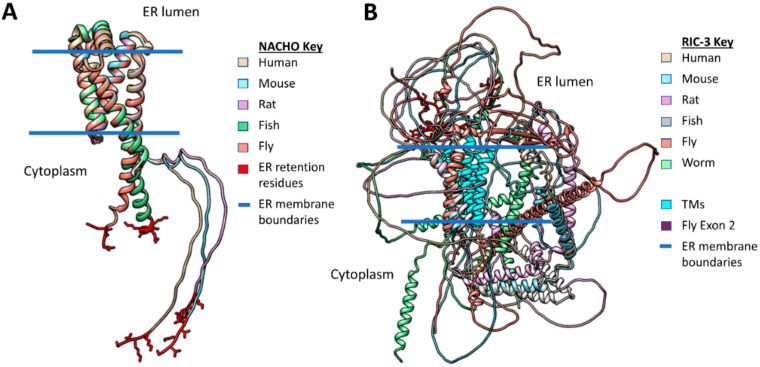
Comparison of full-length Matchmaker-aligned NACHO (Panel (**A**)) and RIC-3 (Panel (**B**)) AlphaFold structures. 4A: NACHO structures coded by colors overlap significantly except for the C-terminal region in the cytoplasm. The C-terminal ER retention residues are shown with side chains in red. The RMSD values for human NACHO (Uniprot Q53FP2) vs. mouse (Q9D328) is 0.13 Ȧ for 138 pruned atom pairs but 5.18 for all 167 pairs, for human vs. rat (Uniprot Q53FP2: 0.19 Ȧ for 135 pruned atom pairs, 7.05 Ȧ for all 167), for human vs. zebrafish (Q53FP2: 0.41 Ȧ for 134 pruned atom pairs, 7.05 Ȧ for all 139), for human vs. Drosophila (Q8T0T9: 0.70 Ȧ for 117 pruned atom pairs, 8.35 Ȧ for all 143). 4B: In contrast, AlphaFold RIC-3 structures diverge significantly in all regions, but aside from alpha helices, all structural predictions have low confidence with high predicted aligned errors. Human RIC-3 (Uniprot Q7Z5B4) vs. mouse (Q8BPM6: RMSD across all 367 pairs: 32.5 Ȧ), vs. rat (B0B1T9: RMSD across all 367 pairs: 21.8 Ȧ), vs. zebrafish (A0A0G2L330: RMSD across all 234 pairs: 34.0 Ȧ), vs. Drosophila (Q9W2N4: RMSD across all 322 pairs: 55.2 Ȧ), and vs. worm (Q22472: RMSD across all 300 pairs: 44.9 Ȧ). Note that AlphaFold does not prohibit non-TM protein strands from crossing the predicted plane of the membrane.

Figure 4 shows AlphaFold predictions for NACHO structures in five species (Figure 4A, Human, mouse, rat, fish, and fly) versus that of RIC-3 (Figure 4B) in the same five species plus *C. elegans*. NACHO structures align almost perfectly using Matchmaker with differences showing only in the cytoplasmic C-terminal regions. The C-terminals contain the ER-retention sequence KVKVS for all the vertebrate species and KQE for the fly (side chains shown in red). All four predicted NACHO TMs overlap for each species with the fly and zebrafish C-terminals continuing as alpha helices, while the mammalian C-terminals are predicted to be unstructured. In contrast, the only structures that AlphaFold predicts with any certainty in RIC-3 are the alpha helices including the putative signal sequences, the TMs, and the coiled-coil domains. Matchmaker aligns the RIC-3 structures so that the signal sequences and TMs are approximately perpendicular to the imagined plane of a membrane, but whether this is coincidence is unclear. AlphaFold shows no certainty in assigning structures for the remaining parts of RIC-3 and non-TM strands readily cross the hypothetical plane of the membrane. As a large, highly flexible molecule, RIC-3 is likely much larger than both NACHO and α7 nAChR on both sides of the ER membrane when fully extended.

**Figure 5:**
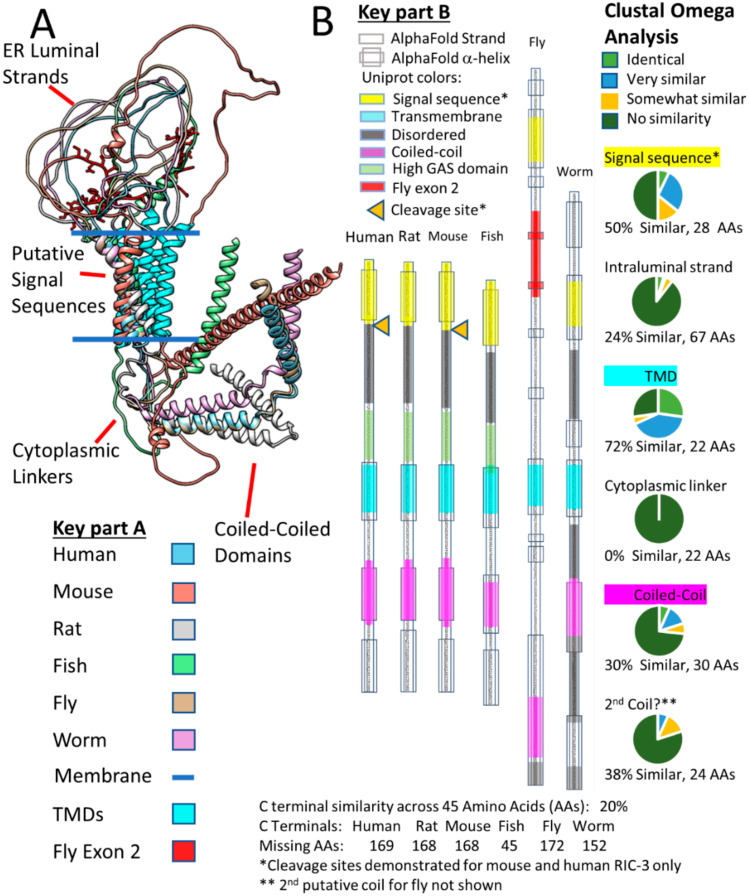
Alignments of pruned RIC-3 species by structures. Panel (**A**) compares AlphaFold PDBs overlayed by UCSF Matchmaker for human, mouse, rat, fish fly and worm RIC-3. The regions before the putative signal sequences and those after the coiled-coil domain(s) are removed for clarity. The putative transmembrane domains (TMDs) are colored cyan for each species to distinguish them from the putative signal sequences shown with the colors corresponding to each species. Fly exon 2 is in red with amino acid side chains shown in the ER intraluminal strand. Panel (**B**) shows the equivalent regions in linear form aligned by the beginning of the Transmembrane Domains (TMDs—shown in cyan) as defined by Uniprot annotation (https://www.uniprot.org (accessed on 21 July 2021)). The putative signal sequences (yellow), disordered sequences (gray), TMDs (cyan), and coiled-coil domains (purple) are colored according to the Uniprot annotation for each gene in addition to high GAS regions (lime) in vertebrate RIC-3s and fly exon 2 (red) in the fly ER intraluminal strand. Alpha-helices predicted by AlphaFold are shown as thicker boxes around the protein strand. C-terminal sequences beyond the second coiled-coil alpha helix (as predicted in the Uniprot annotation for each gene) are not shown and are summarized at the bottom of the figure. The rightmost column of panel (**B**) shows the results of Clustal Omega analysis (https://www.ebi.ac.uk/Tools/msa/clustalo/ (accessed on 21 July 2021)) of the protein sequences. The pie charts for each region show the proportion of identical (light green), very similar (blue), similar (orange), and regions of no similarity (dark green) along with the percentage of total similarity ([identical + very similar + similar amino acids]/total amino acids in the human RIC-3 sequence for that region). The predicted helical regions in Uniprot (Signal sequence, TMD and coiled-coil) align very well with the predicted AlphaFold structures, although AlphaFold predicts some additional short helices in fly and worm RIC-3.

Wang et al. [22] argue that the mouse RIC-3 C-terminal tail past amino acid 181 is dispensable for α7 assembly, and Rex et al. [104] came to a similar conclusion about truncated human RIC-3 lacking amino acids after amino acid 255. Therefore, one way to simplify the RIC-3 structure is to only consider regions starting with the putative signal sequence and ending at the first coiled-coil domain similar to the minimal *C. elegans* RIC-3 discussed by Biala et al. [136]. Figure 5A shows these pruned versions of Alphafold RIC-3 structures from the six species clarifying the important parts of RIC-3 but also demonstrating the diversity of RIC-3 structures in the ER lumen and those connecting the TMD regions to the coiled-coil domains. Isoform C is the AlphaFold structure for Drosophila RIC-3 shown, which according to Lansdell et al. [137] does not support α7 assembly due to the presence of fly exon 2 (Shown in red with amino acid side chains). Fly RIC-3 isoforms lacking exon 2 do support assembly. Note that if the putative RIC-3 signal sequences are cleaved during translation; the ER intraluminal strands of the various isoforms would become even more flexible and can extend farther into the ER lumen. However, signal sequence cleavage (Orange triangles in Figure 5B) is only documented for human [124] and mouse [22] RIC-3 (however see [131]).

Figure 5B compares the predicted alpha-helical regions from AlphaFold with the helical predictions made by Uniprot using other methods, and in general, the regions align well. In addition, Figure 5B summarizes Clustal Omega analysis of the pruned RIC-3 amino acid sequences for the six species. The two regions with the highest sequence similarity across RIC-3 from these six species are in the putative Transmembrane Domain (TMD or the second TM) and the putative Signal Sequence (the first TM) at 72% and 50% similarity respectively. Other regions, in decreasing order of similarity, are a second putative coiled-coil (38%), the first coiled-coil (30%), the ER intraluminal strand between the signal sequence and the TMD (24%) and the cytoplasmic linker between the TMD and the first coiled-coil with no homology (0%) across species. The variable length C-terminals past the second coiled-coil are not shown but ranged between 45 to 178 amino acids and had only 20% similarity by Clustal Omega. Uniprot also predicts that large parts of the different RIC-3 species C-terminals are intrinsically disordered, but Alphafold predicts that a region of the fly C-terminal forms a β-pleated sheet structure (Not shown specifically but buried in the overlap shown in Figure 4B). The regions preceding the TMD in vertebrate RIC-3s (Figure 5B), are rich in glycine, alanine, and serine (High GAS regions, stretches with >70% glycine, alanine or serine), suggesting that these regions are highly flexible with few or short side chains to interact with other proteins. The mystery that emerges from this structure/sequence analysis is how a family of proteins with very little sequence homology except in small regions and almost no defined structure manages to help assemble multimeric receptors. RIC-3 from *C. elegans* assists rat [123] and human [138] while Drosophila RIC-3 (with the appropriate exons) helps human α7 assembly [137].

### 2.4. Two Models for How RIC-3 Helps Assemble α7 Receptors

**Figure 6:**
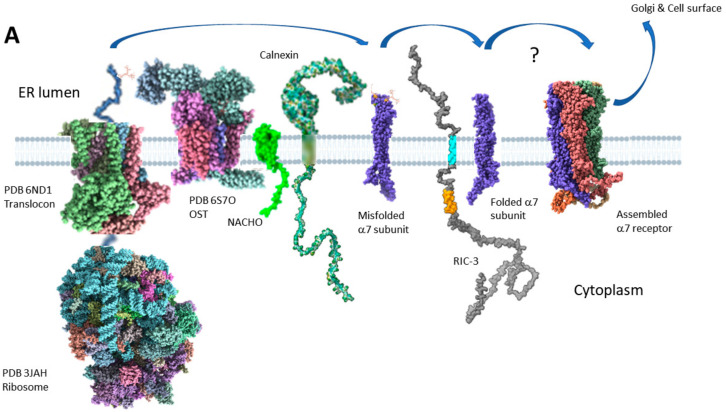

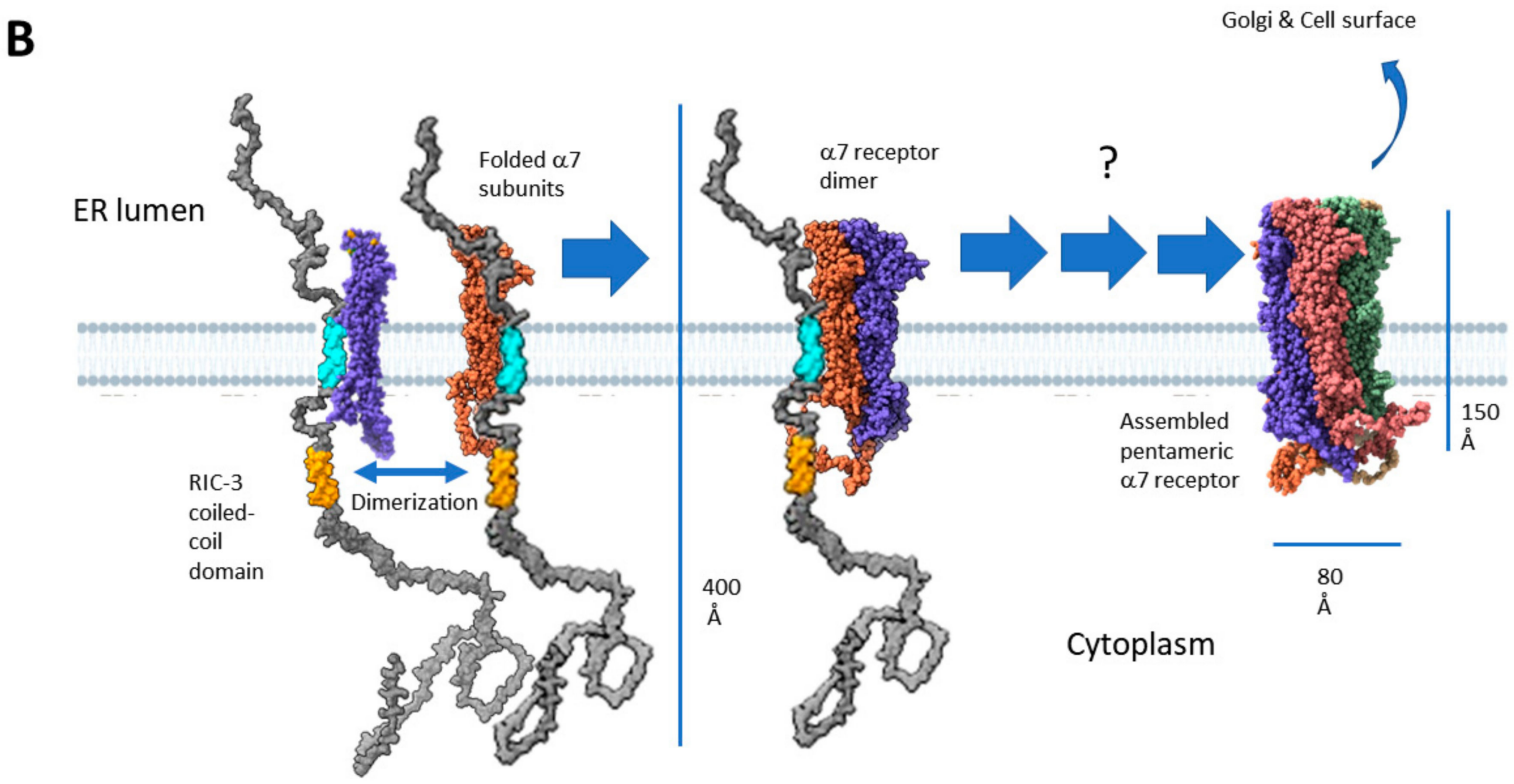
Two models from the literature on how RIC-3 helps assemble α7 nAChRs. Panel (**A**) is based on Kweon et al. (Figure 7D in [23]) and shows cartoons of prominent ER proteins involved in the synthesis, folding and assembly of α7 nAChRs. Cartoons are only approximately to scale and are based on the size of putative transmembrane domains crossing the membrane. The ribosome (PDB 3JAH) is artificially disconnected from the translocon (PDB 6ND1, yeast Sec complex) to show the growing translated α7 chain (skyflower blue) going into the translocon channel. Oligosaccharide transferase (OST PDB 6S7O) attaches high mannose-type glycans to appropriate α7 asparagine residues as the α7 chain emerges from the translocon after removal of the α7 signal peptide. According to Kweon et al. [23], human NACHO (lime green model from AlphaFold AF-Q53FP2-F1) does not directly contact the α7 chain but interacts with both OST (through subunits ribophorins 1 & 2) and Calnexin (Multicolor model from AlphaFold AF-P27824-F1 and PDB 1JHN)). Calnexin interacts with misfolded α7 chains (Purple) through incompletely processed glucose residues in the high mannose α7 cores. RIC-3 (Gray chain from Alphafold AF-Q7Z5B4-F1) has its effects late in the folding process, and properly folded α7 subunits form the basic pentamer that gets further processed in the Golgi apparatus before being trafficked to the cell surface. The putative RIC-3 transmembrane domain is cyan, and the coiled-coil domain is orange. Various phi and psi angles are arbitrarily adjusted in the RIC-3 and calnexin cartoons to prevent disordered protein regions from crossing the membrane. Panel (**B**) is based on Wang et al. (Figure 9 in [22]) This is an older model that precedes the discovery of NACHO. RIC-3 interacts with partially folded α7 subunits, and two α7 subunit-RIC-3 dyads are pulled together through the dimerization of the RIC-3 coiled-coil domains. One of the RIC-3 chains dissociates, leaving the other associated with the partially assembled receptor, and the process repeats with other α7-RIC-3 pairs until the pentamer is formed. The coloring in RIC-3 is the same as in panel (**A**), and various phi and psi angles are arbitrarily adjusted to prevent disordered protein regions from crossing the membrane. The blue lines are distance measurements in Angstroms made by ChimeraX on the models to give an idea of scale. Fully unfolded RIC-3 is many times larger than folded α7 subunits.

Figure 6 shows two models for how RIC-3 might assist α7 receptor assembly. Figure 6A, based on Figure 7D of Kweon et al. [23], primarily shows the effects of NACHO but includes RIC-3 and also many current hypotheses about α7 synthesis and membrane protein folding in general. When the nascent α7 N-terminal emerges from the ribosome, the signal recognition particle aligns the ribosome with the translocon (Sec61 complex, [139,140]), and at some point, the signal peptide is cleaved from the growing chain by signal peptide peptidase [141]. The nascent α7 N-terminal emerges into the ER lumen where it encounters oligosaccharyltransferase (OST), which transfers pre-formed high-mannose glycans from dolichol onto the appropriate asparagines [142], such as N-46, N-90, and N-133 in α7 [143]. Simultaneously, TM alpha helices form in the ribosome tunnel [144] and lead to the insertion into the ER membrane through a lateral opening of the translocon [145]. Kweon et al. [23] cite proteomics data that NACHO does not directly interact with α7 subunits, but instead is associated with ribophorin 1 and 2, two subunits of the OST complex, as well as calnexin [146], another chaperone in the ER. Kweon et al. also show using α7/5-HT3A chimeras that NACHO facilitates the folding of the first and second α7 TM domains over an eight amino acid stretch (See Figure 2A, M2260-7 shown in gray). However, the connection to NACHO interacting with OST and calnexin and how that affects α7 M2 folding is not clear if NACHO doesn’t directly bind to α7 receptors. Calnexin [147,148] (and its soluble colleague calreticulin [149]) recognizes mono-glucosylated N-linked glycans that are present on unfolded glycoproteins and, among other activities, promotes interactions between the unfolded protein and a protein disulfide isomerase ERp57 (also called PDIA3) which itself promotes disulfide bond formation [148] in the unfolded protein. After proper transmembrane folding and disulfide bond formation [150], Kweon et al. propose that the α7 subunits then interact with RIC-3, which helps complete the folding and assembly process without specifying how. α7 nAChRs are also palmitoylated [94,151] and affected by polyamines [152], but these processes are not shown.

Figure 6B proposes that RIC-3 directly binds to partially folded α7 subunits and pairs of α7-RIC-3 dyads pull together by dimerization of the RIC-3 coiled-coil domains. One RIC-3 falls off the resulting tetrad and another α7-RIC-3 pair attaches itself to the growing receptor. This process repeats until a pentamer forms. However, the binding between coiled-coiled domains would necessarily be weak and temporary or else the risk of aggregation ensues. In fact, something like this occurs when the ratio of RIC-3 to α7 subunits is too high [153]. Furthermore, this model requires that RIC-3 has a coiled-coil domain to function. There are natural splice variants of RIC-3 that lack coiled-coil domains and yet promote α7 nAChR assembly [137,154,155,156].

### 2.5. Does RIC-3 Bind to α7 Receptors, and If So, Where?

Wang’s model clearly requires α7 subunits to bind RIC-3, but does it? If, instead, RIC-3 acts as a scaffolding protein to facilitate α7 subunit interactions with other chaperones but does not directly bind to α7 as proposed for NACHO [23], then it raises the question as to how the other chaperones recognize the varied sequences and structures of RIC-3 isoforms across species. However, there is some evidence that RIC-3 binds to α7 nAChR subunits. Human α7 co-immunoprecipitates with human RIC-3 expressed in HEK293 [5] or tsA201 [138]. One report suggests that RIC-3 accompanies α7 nAChRs to the cell surface [5], much like the chaperone stargazin does with glutamate AMPA receptors [134], but other reports suggest that RIC-3 remains exclusively in the ER or possibly can get as far as the Golgi [22,124]. Similarly, various constructs of *C. elegans* RIC-3 co-immunoprecipitate with worm ACR-16 [70]. The proteomics data for RIC-3 association with α7 receptors is murky. α-Bungarotoxin pulled down 121 proteins with α7-nAChRs from wild-type mouse brain, but RIC-3 was conspicuous in its absence (as was NACHO) when compared against the proteins α-bungarotoxin pulled down in α7 knockout animals [157]. An earlier study in mouse brain found similar results [158]. RIC-3 was only marginally detected in proteins identified by α-bungarotoxin pull-down of α7 nAChRs expressed with RIC-3 in the SH-EP1 cell line [159]. The authors state that “This may reflect the fact that Ric-3 is only transiently associated with α7-nAChRs”. However, the protein pull-downs did include other ER resident proteins involved in the early stages of α7 subunit synthesis such as calnexin, calreticulin, dolichol-phosphate mannosyltransferase, and translocon-associated protein subunit gamma. These proteomic results about RIC-3 and α7-nAChRs interactions are reminiscent of the proteomic data suggesting that NACHO doesn’t directly bind α7-nAChRs. However, Wang et al.’s immunoprecipitation data suggest that mouse RIC-3 associates at least in part with partially unfolded α7 subunits that have not yet developed α-bungarotoxin binding [22]. This may make the proteomics data more understandable if partially assembled α7 receptors interacting with RIC-3 only weakly bind α-bungarotoxin and therefore are not easily detected.

#### 2.5.1. RIC-3 Interactions with the α7 Receptor ECD

The ER intraluminal strands proximal to the putative single TMD are the most likely RIC-3 region to interact with the α7-NAChR extracellular domain (ECD), but appear to be poorly suited to the task. For instance, almost half (44%) the TMD-proximal 25 amino acid sequence in human RIC-3 is glycine, and the rest includes 20% alanine and 8% serine (High GAS domain, Figure 5B). This suggests a highly flexible ER intraluminal RIC-3 protein strand with few or short amino acid side chains available for interacting with the α7-nAChR ECD. However, this does not preclude flexible intraluminal sequences located closer to the RIC-3 N-terminal interacting with α7 ECDs. For instance, several Drosophila RIC-3 splice variants promote human α7-nAChR expression in nonpermissive mammalian cells, but splice variants with fly exon 2 suppress expression [137]. Exon 2 is in the middle of the fly RIC-3 ER luminal strand (Figure 5A), and these data suggest that this region does play a role in RIC-3 actions.

Note that in the case of Drosophila RIC-3, there is no evidence that the first TM is a signal peptide as proposed for human [124] or mouse RIC-3 [22]. If the first Drosophila TM is not a signal peptide, then it and the second TM would make Drosophila RIC-3 into a loop in the ER lumen (Figure 5A). However, it is not clear how adding exon 2 and making the loop larger would interfere with α7 folding and assembly. On the other hand, Castelán et al. [131] did not find evidence that human RIC-3 is cleaved by signal sequence peptidase when they translated intact RIC-3 in vitro with microsomal membranes. They did find cleavage if the first TM of human RIC-3 is replaced by the bovine α7 signal sequence, but this alteration decreases RIC-3 efficacy measured by radioactive toxin binding or acetylcholine-evoked ion currents in frog oocytes. Castelán et al. [131] did find that deleting the loop between the first and second TM or replacing it with an irrelevant peptide (derived from the glycine receptor α1 subunit) dramatically decreases RIC-3 efficacy, but that smaller deletions to this loop did not. These data provide more evidence that RIC-3 does show interactions with the ECD of α7 nAChRs.

#### 2.5.2. RIC-3 Interactions with α7 Receptor Transmembrane Domains

The putative single TMD (in vertebrate RIC-3s or the second TM in cases when there is no signal peptide) is the region with the highest probability of interacting with α7-NAChR subunits, as this is the region with the highest sequence conservation across species. Even though BLAST does not find any sequence similarity between fly and human RIC-3, it does find a short piece of similarity between fly and mouse RIC-3 (45 amino acids) that includes the putative TMD (65% identity over 20 amino acids in TMD vs. 33% (15/45) identity overall). Similarly, *C. elegans* RIC-3 is most similar or identical in its sequence to human RIC-3 in the TMD region. Note that Clustal Omega finds even higher similarity in the putative TMD (Figure 5B). Furthermore, mutating the similar TMD amino acids to alanines in *C. elegans* RIC-3 prevents assembly of the worm α7-nAChR-like ACR16 receptor as well as the less similar Deg-3 and Des-2 heteromer [70]. Also, deleting TMD in mouse RIC-3 blocks the assembly of mouse α7-nAChR in BOSC cells [22]. Finally, Castelán et al. [131] report that DNA constructs replacing the putative human RIC-3 TMD with the TM of the EGF receptor show very low efficacy for α7-receptor assembly. RIC-3’s TMD clearly plays a role in its effects.

**Figure 7:**
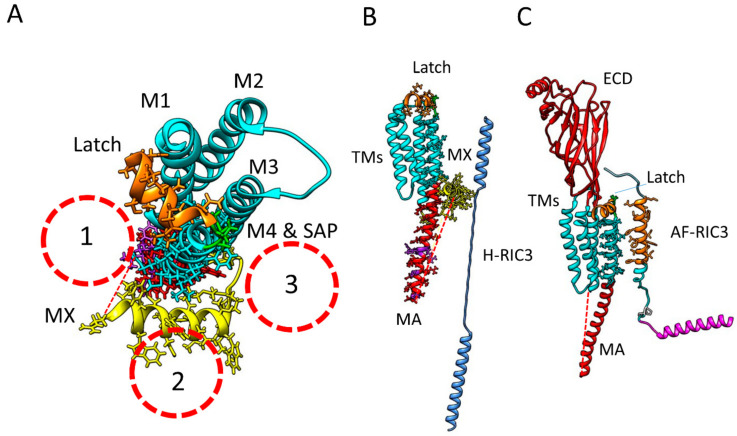
Locations where the RIC-3 transmembrane domain may contact an alpha7 receptor subunit. (**A**). Three locations where the RIC-3 TMD could make contact from a top view. The inside diameters of the dotted circles approximate the size of an alpha helix with the amino acid side chains. (**B**). Side view of how MX would block hyperextended RIC-3 from contacting alpha7-TMs. (**C**). The same view (but including the ECD) if AlphaFold RIC-3 contacted alpha7 M4 before MX is folded into place. A short piece (10 amino acids, 60% glycine) of the RIC-3 intraluminal strand is included at the N-terminal (extracellular) side of RIC-3 TM, and the N-terminal intraluminal remainder could easily contact unknown locations on the extracellular domain of the receptor.

The next question is that if RIC-3 interacts with α7-nAChR subunits in part with its transmembrane domain, which of the receptor TMs does it contact? Although we now know the approximate final locations of α7-nAChR helices in the membrane and cytoplasm, we do not know the order of how they got there during folding and assembly. Furthermore, recent evidence suggests that transmembrane alpha helices form very early in the folding process [144]. Therefore, we might expect RIC-3 to interact with formed α7 TMs. However, if the RIC-3 TMD is contacting α7-nAChR TMs (both structures are restricted to the membrane), it is not likely to contact receptor M1-M3 domains, as doing so places the chaperone in the middle of the receptor being constructed. Looking down on the folded receptor subunit (Figure 7A), there are three likely locations for the RIC-3 TMD helix: (1) in a groove formed by helix MA/TM4 nestled between the latch and MX; (2) contacting MX; or (3) contacting the loop connecting TM3 and MX. Note that in positions 1 and 2 there is a high possibility that the unresolved cytoplasmic loop could interact with RIC-3 in ways we cannot guess at this point. However, on the face of it, none of these locations is ideal. The groove (1) and the loop (3) are complementary in the final assembled pentamer, and occupying these sites with RIC-3 would hinder rather than promote receptor assembly, since RIC-3 would need to be removed before assembly could occur.

However, as shown in Figure 7B, the presence of the MX helix would largely prevent close association between RIC-3 TMD and α7-AChR M4. If these two domains interact, it would be better if their interaction is before the MX helix is put in place (Figure 7C). The MX domains of muscle AChR (α1β1γδ) β and δ subunits are recently proposed to play a role in Golgi retention, ubiquitination and to act as a quality control site in muscle receptor folding [160]. No such role has yet been proposed for the α7 MX domain, but this possibility bears investigation, and as said above, the exact order of structural folding in α7-nAChR is not known. Similarly, a potential ER retention signal in the MA helix has come under scrutiny. Castillo et al. [55,56] performed alanine substitution screens and found five mutations that would not allow enhanced assembly in the presence of RIC-3 (Purple residues in Figure 1): L433A, V440A, R446A, F447A and R448A-numbering as in PDB 7EKI. These residues line up with the MA/TM4 groove (Figure 7A) and may represent an RXR type retention signal that, when exposed, prevents transport from the ER [56,161]. These residues are expected to be covered in a fully assembled α7-nAChR, but it is unclear if RIC-3 contacts these residues, since if it did, the chaperone would likely have to be removed from the groove prior to pentameric assembly.

#### 2.5.3. RIC-3 Interactions with the α7 Receptor Intracellular Domain

The RIC-3 linker regions show low conservation between species and have variable lengths, but the coiled-coil domains appear to be important for function. In *C. elegans*, Deg-3/Des-2 heteromers can assemble with worm RIC-3 lacking the coiled-coil domains [69], but not if TMD is mutated. In contrast, the α7-nAChR-like ACR16 receptor cannot assemble without at least one of the coiled-coiled domains remaining, although the first coiled-coil can be removed [136]. Deletion of either TMD or the coiled-coil domain in mouse RIC-3 blocks receptor assembly [22], and Wang et al. provide evidence suggesting that the coiled-coil is interacting with itself and not directly with α7-nAChR. Further downstream, Wang et al. deleted the c-terminal after the coiled-coil (mRIC3 1-181-myc) and found a significant increase in heterologous α7 receptor expression [22]. Therefore, at a minimum, the consensus seems to be that RIC-3 requires at least the TMD and a coiled-coiled domain with certain unexplained and possibly redundant effects in the N- and C-termini to promote mammalian α7-nAChR folding and assembly in non-permissive cells. The effects in *C. elegans* are more complicated for ACR-16 receptors [136]. Removing the first coiled-coiled domain had no effect on RIC-3’s ability to promote ACR-16 expression in oocytes, but removing the entire C-terminal with all coiled-coil domains largely blocked expression.

Kweon et al. [23] made α7-5HT3 chimeras with the cytoplasmic loop replaced with an α3 cytoplasmic loop sequence, but in these constructs, RIC-3 had little effect on assembly. They interpret these results to show that RIC-3 binds to regions in the cytoplasmic loop, which is certainly possible. However, the effects of RIC-3 on α3β4 heteromers are complicated by the cell expression system. RIC-3 strongly inhibits α3β4 assembly (and several other heteromeric combinations) in oocytes [123] but strongly promotes assembly in HEK tsA201 cells [138]. Therefore, the role of the receptor cytoplasmic loop in the actions of RIC-3 is currently unclear.

### 2.6. RIC-3 Integration with Other Chaperones and Regulators

Multiple studies demonstrate that RIC-3 and NACHO are synergistic in promoting α7 nAChR assembly [6,130,132], suggesting that they act at different steps in the assembly process, as reviewed above. However, these are not the only ER proteins that may help regulate α7 nAChR cell surface expression. Gu et al. [6] found many other genes that, when expressed in HEK cells, promoted increased α7 nAChR-mediated calcium flux in HEK 293T cells, but of those NACHO was the most efficacious. Follow-up studies [54,104] established that many other proteins also affect α7 nAChR cell surface expression. Dawe et al. [54] screened for genes enhancing NACHO’s effects on expression and found several anti-apoptotic Bcl2-like proteins, including Mcl-1 and Bcl-XL (Bcl2: B-cell lymphoma 2, Mcl-1: Myeloid cell leukemia-1 and Bcl-XL: Bcl2-like 1). The co-expression of Mcl-1 or Bcl-XL with α7 and NACHO increases α7 expression over NACHO alone and anti-Bcl2 drugs decrease this effect, but neither protein has an effect on α7 expression by itself. Furthermore, mutating Isoleucine 436 (located in MA in the α7 ICD) to alanine blocks the effects of Bcl2-like proteins on α7 expression (Figure 2A, ICD), suggesting that the site on α7 where the Bcl2-like proteins have their enhancing effects is on the ICD. Finally, lentivirus transfection of Mcl-1 into hippocampal cells boosts alpha-bungarotoxin binding, suggesting that the enhancing effects of Bcl2-like proteins extends to other cell types.

Xenopus oocytes and variants of HEK cells (e.g., 293T, tsa201, BOSC23) are the most common cells for analyzing the effects of chaperones on cys-loop receptor expression, but there are clear cell-type dependent effects. RIC-3 has inhibitory effects on 5HT3A homomeric (but not heteromeric) receptor expression in oocytes [47,126] but enhances expression in monkey COS cells [124,162]. Interestingly, the inhibitory effects of RIC-3 on 5HT3A homo-pentameric expression in oocytes is lost if the ICD is replaced by the bacterial M3-M4 ICD from *Gloeobacter violaceus* [47] and can be induced if a 5HT3A ICD is introduced into *Gloeobacter violaceus* ligand-gated ion channel (GLIC) [163]. These and other data [50,164] suggest that RIC-3 interacts with 5HT3A receptors at least in part through the ICD.

*Drosophila* RIC-3 provides another example of cell-type specific effects. Lansdell et al. [137] report that certain *Drosophila* RIC-3 splice variants promote α7 expression better in a *Drosophila*-derived cell line (S2 cells) than human RIC-3, but the situation reverses when human or *Drosophila* RIC-3 is used in a human cell line (tsA201 cells, a variant derived from HEK 293 cells). These results suggest that no cell type or cell line is a blank slate in terms of the mix of different chaperones present in the ER and that local factors in individual cells can play a big role determining the outcome of receptor folding and assembly. Another factor is that many intrinsically disordered proteins have “moonlighting” functions, meaning that they serve more than one purpose and can bind to multiple proteins involved in very disparate signaling or folding pathways [165]. RIC-3 is a much larger protein than needed for its known effects on Cys-loop receptors, and at this point we can only speculate on what other cellular processes RIC-3 may influence. Finally, RIC-3 itself is regulated. Shteingauz et al. [166] report that BATH-42, a BTB- (broad-complex, Tramtrack and bric-a-brac) and MATH- (meprin-associated Traf homology) domain protein that interacts with proteosomes, also interacts with the RIC-3 C-terminal in *C. elegans* to regulate both RIC-3 and nicotinic receptor expression. Also in *C. elegans*, Safdie et al. [167] report that phosphorylation at RIC-3 Ser164 by calcineurin allows RIC-3 to help regulate worm GABAa receptors, a gain in function that increases nervous system inhibition. How vertebrate RIC-3 expression and function is regulated has not been extensively studied to date.

Finally, this review has focused on chaperone proteins that facilitate α7 nAChR folding and assembly, but there are good reasons to limit the expression of these receptors as well. Homomeric α7 nAChRs are the nicotinic receptors with the highest calcium permeability [168,169,170], and too much calcium influx leads to cell injury or death [171]. Recently, Wu et al. [172] showed that Ly6h antagonizes the effects of NACHO on α7 nAChR assembly in both neurons and HEKtsa cells. Ly6h is a member of the prototoxin Ly6/uPAR protein family [173] that all have three-finger folding structures similar to snake a-neurotoxins such as a-bungarotoxin [25]. Ly6h directly binds to α7 nAChRs [174], and ly6h transfection into hippocampal neurons decreases the α7 responses activated by acetylcholine and PNU120596. One mystery surrounding NACHO is why knocking out the tmem35A gene completely blocks α7 nAChR expression [6,130], while preliminary results from knocking out RIC-3 only causes a slight decrease in mouse brain toxin binding [132]. Based on the ly6h data, it is highly possible that one or several of the Ly6/uPAR members antagonizes NACHO’s effects. However, there are over 60 known Ly6/uPAR family members in the mouse genome [173], suggesting a high possibility of redundancy, so that a single gene knockout may not provide definitive results.

## 3. Summary and Conclusions

Unless someone develops a molecular movie machine that allows atomic-scale visualization of receptor subunit synthesis and folding, we are stuck for the foreseeable future trying to infer the steps involved in receptor folding and assembly from static images of the final products or by making mutations and trying to interpret the consequences. However, the static images of α7 nAChRs are now getting good enough to begin speculating on how the final receptor structure comes together. A major remaining issue for the “resting state” α7 nAChR structure is whether the intracellular loop is highly disordered or has a secondary structure that is free to move in a way that prevents resolution by most current structural methods. Also, it would be useful if a proper bioinformatic search is performed to establish how uniformly the SAP motif is conserved in α7-like nAChRs across species. (Note at least one counter example: The sea anemone *Exaiptasia diaphana* ACR-16 {Accession XP_028515061] has an SSP motif, but this also raises complicated issues about inclusion criteria for being “a7-like”.) The question would be more interesting if it could be established whether the SAP motif is important for receptor function or assembly.

The utility of AlphaFold to predict structure for highly disordered proteins such as RIC-3 is almost a contradiction in terms, but with proper adjustments, AlphaFold allows for the visualization of how the various parts of RIC-3 and α7-nAChR subunits might interact. In addition, the Uniprot predictions for RIC-3 alpha helical structure correlate closely with those of AlphaFold.

Wang’s hypothesis that RIC-3’s coiled-coil domains dimerize to recruit bound α7-nAChR subunits into the growing pentamer is conceptually appealing, but the counter-examples of functional RIC-3 splice variants lacking a coiled-coil domain suggest that if the coiled-coil domains act that way, there must be redundant effects elsewhere in the RIC-3 molecule. We still do not have a coherent view of what RIC-3 or NACHO do to promote α7-nAChR assembly.

## 4. Methods

Although this is largely a review, various programs were used to analyze available data. USCF Chimera [175] or ChimeraX [176] rendered protein structure models, The Resource for Biocomputing, Visualization, and Informatics at the University of California, San Francisco developed ChimeraX with support from the National Institutes of Health R01-GM129325 and the Office of Cyber Infrastructure and Computational Biology, National Institute of Allergy and Infectious Diseases. Matchmaker [177] (a subprogram within Chimera and ChimeraX) aligned protein PDB files and calculated root mean square deviations (RMSDs). NCBI BLAST [178] and Clustal Omega [179] aligned protein sequences and provided estimates of sequence homologies. Swiss-Model software [180,181,182] (swissmodel.expasy.org) generated homology models based on amino acid sequences. The UniProt website (www.uniprot.org/ (accessed on 21 July 2021)) provided predictions for TMs, coiled-coil domains and disordered regions in addition to linking to the AlphaFold database (https://alphafold.ebi.ac.uk/ (accessed on 21 July 2021)). Uniprot uses SignalP v.3 [183] to detect signal peptides, Coils v.2.2 [184] for predicting coiled-coil domains and a variety of software to predict transmembrane domains [185,186,187]. The Uniprot website did not specify which software predicted intrinsically disordered protein sequences, but https://iupred2a.elte.hu/ (accessed on 10 March 2022) [188] verified the Uniprot website predictions. The RCSB PDB database (https://www.rcsb.org/ (accessed on 8 December 2020)) provided molecular structures based on experimental data. Amino acid numbering in α7 nAChRs is based on the sequence in PDB 7EKI which includes the signal peptide.

## Data Availability

Not applicable.

## Abbreviations

AMPA: alpha-amino-3-hydroxy-5-methyl-4-isoxazole propionic acid. M1–M4: nAChR TMs 1–4. NACHO: Nicotinic acetylcholine receptor regulator, nAChR: Nicotinic Acetylcholine Receptor, PDB: Protein Data Base file RIC-3: Resistance to inhibitors of cholinesterase-3, RMSD: Root mean square deviation, TM: Generic transmembrane domains, TMD: The putative single TM for RIC-3 species that have a signal peptide (TM2 for those that do not), TMEM35A: Transmembrane protein 35, also known as the gene for NACHO.
